# High Affinity Human Antibody Fragments to Dengue Virus Non-Structural Protein 3

**DOI:** 10.1371/journal.pntd.0000881

**Published:** 2010-11-09

**Authors:** Nicole J. Moreland, Moon Y. F. Tay, Elfin Lim, Prasad N. Paradkar, Danny N. P. Doan, Yin Hoe Yau, Susana Geifman Shochat, Subhash G. Vasudevan

**Affiliations:** 1 Program in Emerging Infectious Diseases, DUKE-NUS Graduate Medical School, Singapore, Singapore; 2 School of Biological Sciences, Nanyang Technical University, Singapore, Singapore; Tropical Medicine Institute Pedro Kourí (IPK), Cuba

## Abstract

**Background:**

The enzyme activities catalysed by flavivirus non-structural protein 3 (NS3) are essential for virus replication. They are distributed between the N-terminal protease domain in the first one-third and the C-terminal ATPase/helicase and nucleoside 5′ triphosphatase domain which forms the remainder of the 618-aa long protein.

**Methodology/Principal Findings:**

In this study, dengue full-length NS3 protein with residues 49 to 66 of NS2B covalently attached via a flexible linker, was used as bait in biopanning with a naïve human Fab phage-display library. Using a range of truncated constructs spanning the NS2B cofactor region and the full-length NS3, 10 unique Fab were identified and characterized. Of these, monoclonal Fab 3F8 was shown to bind α3″ (residues 526 through 531) within subdomain III of the helicase domain. The antibody inhibits the ATPase and helicase activites of NS3 in biochemical assays and reduces DENV replication in HEK293 cells that were previously transfected with Fab 3F8 compared with mock transfected cells.

**Conclusions/Significance:**

Antibodies such as 3F8 are valuable tools for studying the molecular mechanisms of flaviviral replication and for the monospecific detection of replicating dengue virus *in vivo.*

## Author Summary

Dengue virus is the most prevalent mosquito transmitted infectious disease in humans and is responsible for febrile disease such as dengue fever, dengue hemorrhagic fever and dengue shock syndrome. Dengue non-structural protein 3 (NS3) is an essential, multifunctional, viral enzyme with two distinct domains; a protease domain required for processing of the viral polyprotein, and a helicase domain required for replication of the viral genome. In this study ten unique human antibody fragments (Fab) that specifically bind dengue NS3 were isolated from a diverse library of Fab clones using phage display technology. The binding site of one of these antibodies, Fab 3F8, has been precisely mapped to the third α-helix within subdomain III of the helicase domain (amino acids 526–531). The antibody inhibits the helicase activity of NS3 in biochemical assays and reduces DENV replication in human embryonic kidney cells. The antibody is a valuable tool for studying dengue replication mechanisms.

## Introduction

Dengue virus belongs to the *Flaviviridae* family and is the etiological agent of dengue fever, dengue hemorrhagic fever and dengue shock syndrome. It is the most prevalent arthropod transmitted infectious disease in humans and has four antigenically distinct viral serotypes (DENV 1–4) [1]. The genome of dengue viruses comprises a positive single stranded RNA of 11kb. Post-translational processing of the polyprotein gives rise to three strucural proteins (C, prM and E) and seven non-structural proteins (NS1, NS2A, NS2B, NS3, NS4A, NS4B and NS5). The processing of the amino terminal region of the polyprotein is carried out by host signal peptidases, while processing of the 2A-2B, 2B-3, 3-4A and 4B-5 sites is catalysed by the two-component viral protease NS2B/NS3 [2], [3].

**Figure 1:**
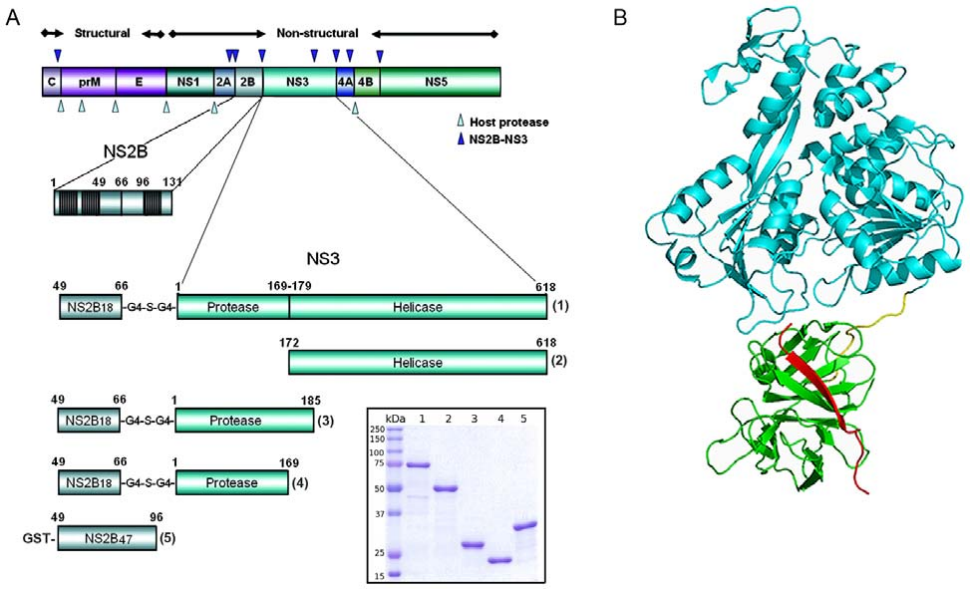
The overall structure of Dengue Non-structural Protein 3. (A) Dengue polyprotein organization and the NS3 protein constructs used in this work. Proteolytic sites targeted by proteases from the host cell and by NS2B-NS3 are indicated with light and dark blue triangles, respectively. The three predicted membrane-associated regions within the NS2B proteins are represented as filled boxes. In the full-length and protease domain constructs residues 49 to 66 of the NS2B protein were linked to the N-terminus of NS3 via a Gly_4_-Ser-Gly_4_ linker, while residues 49 to 96 were linked to GST in the NS2B_47_ construct. The insert shows SDS-PAGE of the purified DENV4 NS3 proteins. Lane numbering (1–5) corresponds with construct numbering in the schematic. (B) The structure of DENV4 NS2B_18_NS3 [8]. The helicase domain is shown in green, the protease domain in cyan and NS2B_18_, which forms a β-strand, is in red.

DENV NS3 is a multifunctional enzyme with three known catalytic activities segregated into two distinct domains (Figure 1). The serine protease lies within the N-terminal 180 amino acid residues of the 618 amino acid protein. The central hydrophillic portion of the intergral membrane protein NS2B (residues 49–96) is required for protease activity [4]–[6]. The ATPase/helicase and nucleoside 5′-triphosphate activities are localised in the remaining C-terminal domain. There appears to be cross-talk between the two domains; the helicase activity is approximately 30-fold higher in the full-length NS3 protein than in the domain and the affinity of the full-length protein for ATP is 10 fold lower than that of the helicase domain alone [7], [8]. Recent crystal structures of full-length NS3 from DENV and the related flavivirus Murray Valley encephalitis virus, reveal that the protease and helicase domains are linked by an interdomain linker (residues 169–179 in DENV) as illustrated in Figure 1 [8], [9].

Infection with one DENV serotype results in immunity to that serotype only; this protection is thought to be due to neutralizing antibodies, DENV-specific memory T cells, or a combination of the two. While the T-cell response is directed against several DENV proteins, NS3 appears to be the dominant target for CD4+ and CD8+ T cells, and multiple human T cell epitopes have been mapped onto NS3 (reviewed in [10]). Interestingly DENV NS3 also elicits a specific antibody response in humans. A study of acute sera from patients infected with DENV-2 or DENV-4 showed that although anti-E (envelope) antibodies were the most abundant, anti-NS3 antibodies were widely detected, particularly in those with secondary infections [11].

Given the vital role NS3 plays in viral replication, and the specific T- and B-cell responses observed towards NS3 in DENV infected patients, well characterised anti-NS3 antibodies would be vaulable tools for studying viral replication in detail and detecting DENV infected cells. There are very few reports describing the production of monoclonal antibodies specific for NS3, and those that have used hybridoma technology in mice [12]–[15]. In three of these studies anti-NS3 antibodies were isolated following immunization with recombinant NS3 [12]–[14] but the fourth study innoculated mice with DENV-1 virus (purified from suckling mouse brain) and selected hybridomas that produced anti-NS3 antibodies [15]. These antibodies were then used to immunize mice that were subsequently challenged with DENV-1. Intriguingly an increase in survival, albeit equivocal, was noted with four of the monoclonal antibodies tested.

Recombinant antibody technology (phage display) is a powerful alternative to conventional antibody techniques that permits the selection of high-affinity antibodies specific for the target protein [16], [17]. Antibody fragments are expressed on the surface of filamentous phage linking the antibody protein with its encoding DNA sequence within the phage. Phage displaying antibodies that bind the target protein are enriched by several rounds of selection and amplification (bio-panning), and the resulting antibody fragments can be produced recombinantly in *E. coli* without the need for immunization. This study describes the identification and characterisation of human Fab antibody fragments that bind DENV NS3 using phage display. The panel of antibodies have different specificity patterns towards the NS3 protease and helicase domains, and NS3 proteins from DENV 1–4. We have evaluated the ability of the antibodies to inhibit the protease, helicase and ATPase activities catalysed by NS3 *in vitro* and DENV replication using cell based assays, and have identified one Fab, designated 3F8, that recognises a conserved epitope on subdomain III of the NS3 helicase domain. This antibody is cross-reactive with all four serotypes, and binds NS3 with high affinity. It can be used as a tool to study the DENV replication complex or could potentially be developed as a therapeutic.

## Materials and Methods

### Cloning and expression of recombinant NS3 domains

A schematic of the constructs used in this study is shown in Figure 1A. The pET32b expression contructs for DENV4 NS2B_18_NS3 full-length (NS3 protein residues 1-618 linked with residues 49–66 of NS2B via a Gly_4_-Ser-Gly_4_ linker) and DENV4 NS3 helicase (residues 172–618) have been described previously [8], [18]. Expression constructs for DENV1-3 pET32b NS2B_18_NS3 full-length proteins were kind gifts from the Novartis Intitute for Tropical Diseases, Singapore, and comprise the same corresponding residues as the DENV4 full length construct.

The DENV4 protease domain constructs were amplified from the DENV4 NS2B_18_NS3 full-length construct. For DENV4 NS2B_18_NS3pro_169_ (NS3 protein residues 1–169 linked with residues 49–66 of NS2B via a Gly_4_-Ser-Gly_4_ linker) the forward primer 5′-CCACGCGGTTCTCATATGGCAGACTTGTCACTA-3′ and the reverse primer 5′-TTCATAATCTGGATCCCCAATTCATTCAGCTTGCGT-3′ were used. The underlined sequence corresponds with the *Nde*I and *Bam*HI sites, respectively. DENV4 NS2B_18_NS3pro_185_ (NS3 protein residues 1–185 linked with residues 49–66 of NS2B via a Gly_4_-Ser-Gly_4_ linker) was amplified using the same forward primer as above and the reverse primer 5′-GTAAGTCCATTATGGATCCTCTTTACTTTCGAAAAATG-3′. The PCR fragments were digested with *Nde*I and *Bam*HI and cloned into pET14b (Novagen). *Escherichia coli* BL21 Codon Plus (DE3)-RIL cells (Stratagene) transformed with pET32b or pET14b expression constructs were grown in ZYM5052 autoinduction medium [19] for 4 hours at 37°C followed by 20 hours at 18°C.

The PCR fragment for the DENV4 GST-NS2B_47_ expression construct (encoding residues 49–96 of NS2B fused at the N-terminus with Glutathione S-transferase) was generated with the forward primer 5′-GTGGTGGATCCGCAGATCTGTCACTAGAG-3′ and reverse primer 5′-CAGTGAATTCAAAAGTCATATCATATTGGTTTCCTC-3′ (*Bam*HI and *Eco*RI sites are underlined) and the previously constructed DENV4 pET15b CF47-NS3 protease vector [6] as template. The PCR product was digested with *Bam*HI and *Eco*RI and cloned into pGEX-4T-1 (GE Healthcare). *E. coli* BL21 Codon Plus (DE3)-RIL cells transformed with the DENV4 GST-NS2B_47_ construct were grown in in ZYM5052 autoinduction medium at 37°C for 4 hours followed by 16°C for 20 hours.

### Protein purification

The DENV1-4 NS2B_18_NS3 full-length and DENV4 NS3hel proteins were purified by immobilised metal-ion affinity chromatography (IMAC) and size exclusion chromatography (SEC) as described previously [8], [18]. SEC was performed in 20 mM Tris pH 7.5, 150 mM NaCl, 3 mM β-mercaptoethanol and 5% glycerol. The pET14b DENV4 NS3 protease proteins were purified using the same protocol except the SEC buffer was 20 mM Hepes pH 7.5, 250 mM NaCl, 3 mM β-mercaptoethanol and 5% glycerol.

DENV4 GST-NS2B_CF47_ cell pellets were lysed in 20 mM Tris pH 7.5, 200 mM NaCl and the clarified lysate was incubated with Glutathione Sepharose 4B (GE Healthcare) for 2 hours at 4°C. Beads were washed extensively in lysis buffer and the protein was eluted in a buffer containing 20–50 mM reduced glutathione followed by dialysis into 20 mM Tris pH 7.5, 200 mM NaCl and 1 mM β-mercaptoethanol for storage.

### Biotinylation of DENV4 NS2B_18_NS3 full length

Purified DENV4 NS2B_18_NS3 full length was dialysed into phosphate buffered saline (PBS) pH 7.5 prior to biotinylation. The protein was incubated with a 20-fold molar excess of the biotin reagent on ice for 2 hours according to the manufacturers instructions (Thermo Fisher Scientific). The reaction was stopped with 100 mM glycine and excess biotin was removed by SEC in PBS pH 7.5.

### Phage display Fab library screeing

Library screening was performed with a naïve human fab phage display library HX02 (Humanyx Pte Ltd, Singapore) displayed in a modified pCES1 vector [20]. The amber stop codon prior to bacteriophage gene III in pCES1 has been removed and replaced with a *Sal*I site. An additional *Sal*I site has been placed at the C-terminus of gene III such that following *Sal*I digestion and religation (and the concurrent formation of a TAA stop codon) soluble Fab can be expressed in both suppressor and non-suppresor strains of *E. coli*.

Library panning was essentially performed as decribed previously [21] but streptavidin megnetic beads (Invitrogen) were used to immobilise the antigen (biotinylated DENV4 NS2B_18_NS3). The concentration of DENV4 NS2B_18_NS3 was 200 nM in the first round and reduced to 40 nM and 10 nM in rounds two and three, respectively. The number of input phage in each round was constant at 1×10^12^ pfu while washing was increased from six times with PBS-T (0.1% Tween-20) in round one to 14 times with PBS-T in rounds two and three. Bound phage were eluted with 100 mM triethylamine and used to infect *E. coli* TG1 cells. Phage were resuced with M13K07 helper phage and amplified on 2xTY (tryptone-yeast) agar plates supplemented with 100 µg/mL ampicillin and 25 µg/mL kanamycin. Plates were scraped with Tris-buffered saline (TBS) and phage was concentrated from the supernatant by polyethylene glycol-NaCl precipitation.

Following the third round of selection individual TG1 clones were rescued with M13K07 and screened by enzyme-linked immunosorbent assay (ELISA) for reactivity against DENV4 NS2B_18_NS3 full-length (coated at 5 µg/mL in PBS pH 7.5). An anti-M13-horse radish peroxidase (HRP) conjugate (GE Healthcare) was used for detection and clones with an absorbance value two times higher than background levels were considered positive. To assess clone uniqueness a *Bst*N1 restriction digest was performed following PCR amplification of the Fab coding region of the phagemid. Clones with unique DNA fingerprints were subject to automated sequencing.

### Expression, purification and Elisa of Fab fusion proteins

Phagemids from unique Fab-phage clones were digested with *Sal*I to remove the gene III coding sequence and re-ligated with T4 DNA ligase. The resulting plasmids were transformed into *E. coli* Top10 F' cells (Invitrogen) for expression and periplasmic extraction. Cell pellets were resuspended in chilled lysis buffer (120 mM Tris pH 8.0, 0.3 mM EDTA and 300 mM surose) and incubated on ice for 30 minutes for periplasmic extraction. Magnesium chloride (2.5 mM) was added to the clarified extract prior to IMAC purification. Fab were further purified by SEC (S200 10/300 column) if required.

For ELISA Maxisorb Immunoplates (Nunc) were coated with the relevant NS3 antigen (0.25 µM) in PBS pH 7.5 and blocked with 5% skim milk in PBS-T. Blocked wells were incubated with purified Fab (100 nM unless otherwise stated) at room temperature for 1 hour. Plates were washed with PBS-T and incubated with an anti-c-*myc* HRP conjugate (Roche) for detection.

### Measurement of binding affinities

Binding affinities of the Fab for DENV4 NS2B_18_NS3 were determined by surface plasmon resonance (SPR) using a Biacore 3000 instrument (GE Healthcare). All experiments were conducted at 25°C in HBS-EP (10 mM Hepes, 150 mM NaCl, 3.4 mM EDTA, 0.0005% P-20, pH 7.4). Biotinylated full-length NS3 protein was captured on a streptavidin (SA) sensorchip at a flow rate of 10 µl/min. For screening, the 10 Fab (100 nM) were injected across the flowcells, in replicates, at 10 µl/min for 1 min and allowed to dissociate for 2.5 min. Regeneration of the surface was achieved by a 30 second pulse with 15 mM HCl. The Fab that showed the best apparent KD in screening were selected for kinetic analysis. Kinetic parameters were measured by varying the molar concentration of each Fab (3.9–500 nM) and injecting these across the flowcells, in duplicates, with the same conditions used in the screening. Raw sensorgram data were aligned, solvent-corrected and double-referenced using the Scrubber II software (BioLogic Software, Campbell, Australia). A simple 1∶1 model, with or without the mass transport coefficient, was used for global kinetic analysis as appropriate.

### Enzyme assays

NTPase assays were conducted as previously decribed [7]. DENV4 NS2B_18_NS3 full-length (4.8 nM) was preincubated with 1 µM Fab for 30 minutes at room temperature in 90 µl of reaction buffer (50 mM Tris pH 7.4, 2 mM MgCl_2_, 1.5 mM DTT, 0.05% Tween 20, 0.25 ng/µl bovine serum albumin). Poly U (1 µg, average length 200–250 bases) was added and a further 5 minute incubation at 37°C was performed before initiating the reaction with 10 µl of ATP. The reaction was carried out at 37°C for 30 minutes after which the malachite green reagent (Sigma) was added and absorbance (630 nm) was measured. The amount of phosphate released was determined with a standard curve and all assays were carried out in triplicate.

Protease activity was determined for NS2B_47_NS3pro_185_ (NS3 protein residues 1–169 linked with residues 49–96 of NS2B via a Gly_4_-Ser-Gly_4_ linker) based on protocols published by Li *et al.* [6] as detailed in the supporting information (Figure S1 in Supporting Information S1).

Helicase activity assays were performed as published [7], [22]. The substrate was prepared by annealing an 18-mer DNA oligo (5′-GCCTCGCTGCCGTCGCCA-3′) with a 38-mer RNA oligo (5′-UGGCGACGGCAGCGAGGCUUUUUUUUUUUUUUUUUUUU-3′). The 5′ end of the DNA was labelled using T4 polynucleotide kinase and [γ−^32^P]ATP. Reactions (10 µl) contained 50 mM Tris-HCl pH 7.4 supplemented with 5 nM of the DNA:RNA duplex, 500 nM DENV4 NS2B_18_NS3 full-length, 1.75 µM Fab (NS3:Fab ratio 1∶3.5), 4 units of RNAsin, 2 mM MgCl_2_, 1 mM DTT, 0.5% Tween and 0.25 µg/mL BSA. Fab and NS3 were preincubated in assay buffer for 10 minutes prior to initiating the reaction with 5 mM ATP. Assays were performed at 37°C for 30 minutes and were resolved on a 10% native polyacrylamide gel and autoradiographed using a Pharos FX system. Signal intensity was quantified with Quantity One software (Biorad).

Statistical analysis of all assay data was performed using paired t-tests. The results were considered statistically significant if p<0.05.

### Cell based assays

HEK293 cells were maintained at 37°C in a CO_2_ incubator in Dulbeco's modified eagle's medium (DMEM) containing 10% fetal calf serum and 1% penicillin-streptomycin. DENV2 (Eden 3295) was propagated in C6/36 cells prior to infection. For Fab transfection, 5×10^4^ cells per well were transfected with 1 µg Fab (3F8, or the non-NS3 binding control Fab 3F6) using the TurboFect protein transfection reagent (Fermentas) and control cells were mock-transfected with TurboFect according to the manufactures instructions. Cells were infected four hours post transfection with DENV2 (Eden 3295) at an MOI of 1.0 in fresh media. For immunofluorescence cells were fixed 48 hours post infection using methanol, and incubated with 4G2 mouse monoclonal antibody for two hours at room temperature followed by a goat-anti-mouse secondary antibody conjugated with Alexa-488. Coverslips were mounted using ProLong Gold antifade reagent with DAPI (Invitrogen). Cells were visualized by fluorescence microscope using the 20X objective.

For plaque assay, media supernatants were collected 48 hours post-infection and virus titers (plaque forming unit per ml, PFU/ml) were determined by a plaque assay on BHK-21 cells as previously described [23]. Western bots were performed using the 4G2 mouse monoclonal antibody and an anti-His-tag antibody. Anti-GAPDH antibody was used as a loading control.

### Epitope mapping of 3F8 using peptide phage display

The Ph.D-12 random dodecapeptide library was purchased from New England Biolabs. Panning was performed as described in the New England Biolab Instruction Manual. Purified 3F8 (240 pmol) was mixed with 1×10^11^ pfu phage for 20 minutes at room temperature. Phage that bound 3F8 were isolated using anti-*c-myc* resin (Thermoscientific). Resin was washed 10 times and bound phage were eluted with 200 mM Glycine-HCl pH 2.2. The amplified eluate was enriched by two further rounds of selection. To minimize target unrelated peptides, phage that bound 3F8 were isolated using magnetic Ni-NTA agarose beads (Qiagen) in the second round. In the third round phage were pre-incubated with anti-*c-myc* resin and Ni-NTA magnetic beads in a ‘negative’ selection step prior to incubation with 3F8. Individual phage clones were purified following the third round of biopanning and tested for reactivity in an ELISA with an anti-M13-HRP conjugate. Single stranded DNA was isolated from positive clones using an iodine buffer (10 mM Tris pH 8.0, 1 mM EDTA, 4 M NaI) and sequenced using the M13 (-96gIII) primer provided in the library kit.

The epitope identified by peptide phage display was verified by competition ELISA using an array of overlapping 15-mer peptides purchased from Mimotopes that span subdomain III of the NS3 helicase domain (DENV-2 strain 16681). 3F8 at a concentration of 0.6 nM was preincubated with 3 nM of peptide for 30 minutes at room temprature before being transferred to an immunoplate previously coated with DENV2 NS2B_18_NS3 (5 µg/mL in PBS pH 7.5). For control, full-length DENV2 NS2B_18_NS3 was used as a competing reagent. Bound Fab was detected with an anti-c-*myc*-HRP conjugate and all measurements were repeated in duplicate and the mean value taken.

## Results

### Identification of anti-NS3 Fab

The NS3 full-length proteins (DENV 1–4) and the DENV4 NS3 domain constructs were purified at yields of between 5–10 mg/litre of culture (Figure 1A). DENV4 NS2B_18_NS3 was biotinylated using a 20 fold excess of the biotin reagent, bound onto streptavidin magnetic beads, and used to screen for binding against the naïve human Fab phage library. After three rounds of selection 480 TG1 clones were screened for their ability to bind DENV4 NS2B_18_NS3 by ELISA. A *Bst*N1 digest of the 150 positive clones enabled grouping of clones with similar DNA fingerprints. Sequencing confirmed the identification of 10 unique clones. Sequence analysis with IMGT/V-QUEST showed that all the variable heavy chain (V_H_) sequences belong to the VH3 or VH4 gene families while the variable light (V_L_) sequences selected are derived from a larger number of gene families (Vκ2, Vκ3, Vλ1, Vλ2, Vλ6) (Table S1 in Supporting Information S1). The heavy chain complementary determining region 3 (CDR3) has been shown to have the most influence over antibody binding specificity [24]. CDR3 heavy chain sequences of the anti-NS3 clones selected are diverse in length and composition.

### Domain specificity and cross-reactivity of the unique Fab

**Figure 2:**
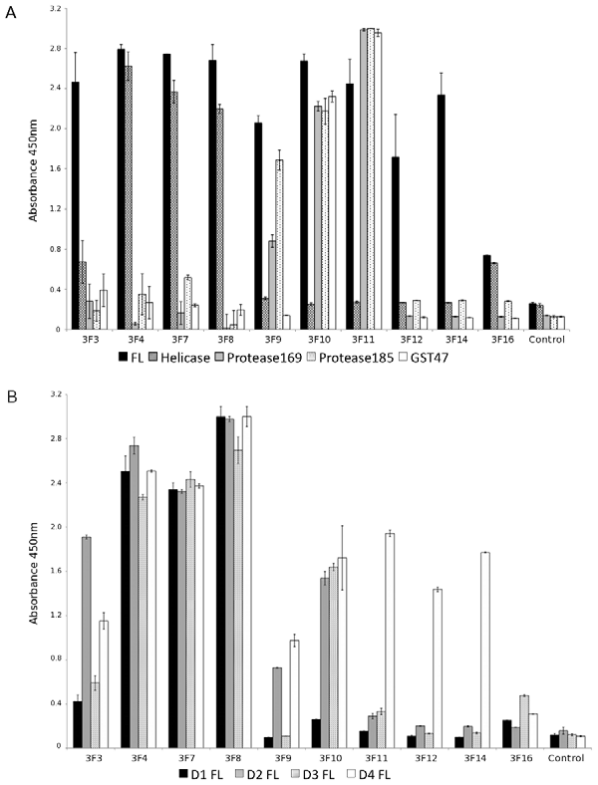
Binding specificity of the Fab (100 nM) as measured by ELISA. (A) Comparison of Fab binding to DENV4 NS2B_18_NS3 full-length (black), DENV4 NS3 helicase (cross-hatch), DENV4 NS2B_18_NS3pro_169_ (grey), DENV4 NS2B_18_NS3pro_185_ (dots) and DENV4 GST-NS2B_47_ (white). (B) Comparison of Fab binding to NS3 full-length proteins from all four dengue virus serotypes; DENV-1 (Hawaii, black), DENV-2 (TSV01, grey), DENV-3 (S22103, cross-hatch) and DENV-4 (MY22713, white). An unrelated Fab is included as control.

The phagemids of the 10 unique clones were digested with *Sal*I to remove the gene III sequence and enable expression of the soluble Fab in *E. coli* Top 10F' cells. The expressed Fab have a hexa-histidine and *c-myc* tag at the C-terminus of the C_H_ domain and were purified from the periplasm of Top10 F' *E. coli* cells by IMAC. Binding of the Fab to the antigen used in panning (DENV4 NS2B_18_NS3 full-length) and the DENV4 NS3 domain constructs was confirmed in an ELISA incorporating 100 nM Fab and an anti-*c-myc*-HRP conjugate (Figure 2A). Three Fab clearly bind the helicase domain of NS3 (3F4, 7 and 8), while a further two Fab (3F3 and 16) also appeared to bind the helicase domain, although the signal at 100 nM was low. An ELISA using 1000 nM Fab confirmed the helicase specificity of 3F3 and 16 (data not shown). Two Fab (3F10 and 11) gave positive signals with DENV4 NS2B_18_NS3pro_169_, DENV4 NS2B_18_NS3pro_185_, and DENV4 GST-NS2B_47_ indicating they bind to the 18 residues of NS2B (49 through 66) required for maintaining stability of the protease domain as these are the only residues present in all three domain constructs. 3F9 binds both DENV4 NS2B_18_NS3pro_169_ and DENV4 NS2B_18_NS3pro_185_ but has a higher signal with the construct ending at residue 185. This suggests the epitope for 3F9 spans the protease domain (up to residue 169) and residues 170 through 185. X-ray crystallography data shows that residues 169–179 form the 10-residue linker located between the protease and helicase domain in the DENV4 NS3 full-length structure and 3F9 may bind this linker region (Figure 1B). The two remaining Fab (3F12 and 14) bind only the the full-length NS3 protein. The epitopes for these Fab maybe structural epitopes located at the interface of the protease and helicase domains, that are only present in the full-length protein.

An ELISA performed with NS2B_18_NS3 full-length from all four DENV serotypes and 100 nM Fab (Figure 2B) showed that three of the helicase specific Fab (3F4, 7 and 8) are cross-reactive with all the serotypes tested. 3F10 binds DENV-2, DENV-3 and DENV-4, while 3F3 and 3F9 bind DENV-2 and DENV-4. Three Fab (3F11, 12 and 14) were DENV-4 specific. The signals for 3F16 are low but indicate it cross-reacts with DENV-3 and DENV-4.

### Binding affinities of Fab to DENV4 NS3

**Figure 3:**
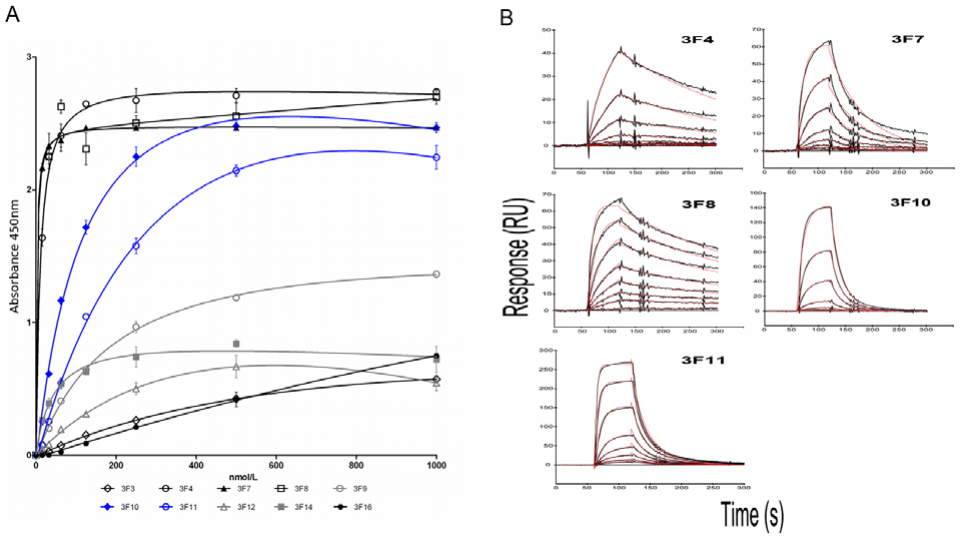
Binding affinity of the Fab to DENV4 NS2B_18_NS3. (A) An ELISA with serial dilutions of Fab starting from 1000 nM. (B) Kinetic analysis of the Fab at 3.9–500 nM using surface plasmon resonance (black). Aligned, solvent-corrected and double-referenced responses were globally fit to a simple 1∶1 binding model (red).

An ELISA using serial dilutions of the purified Fab showed that all Fab gave concentration-dependent binding curves with 3F4, 7, 8, 10 and 11 recognising NS3 at concentrations of less than 100 nM (Figure 3A). 3F8 in particular has a high ELISA signal at 15 nM which suggests a high binding affinity. 3F3, 9, 12, 14 and 16 appear to bind to NS3 with lower affinity.

To further probe the affinity of the Fab for NS3, kinetic rates and affinity constants were measured in real-time using SPR. Biotinylated DENV4 NS2B_18_NS3 was immobilised on a SA sensor chip and, for initial screening, all 10 Fab were injected at a concentration of 100 nM. Binding responses were observed for five of the Fab at 100 nM (3F4, 7, 8, 10 and 11), while the remaining Fab showed no binding at 100 nM (3F3, 9, 12, 14, 16). The five Fab that showed a binding response also gave the highest signals in the affinity ELISA against NS3 (Figure 3A). Kinetic studies were performed on the binding Fab over a concentration range of 3.9–500 nM (Figure 3B). The Fab ranged 70-fold in their affinity for DENV4 NS3, with the highest affinity observed for 3F8 (K*_D_* 10.5 nM). This was followed by 3F7 (94.9 nM), 3F11 (95.6 nM) and 3F4 (98 nM). 3F10 had the lowest affinity of those measured with a K*_D_* of 670 nM (Table 1). This contrasts with the ELISA binding curve where 3F10 reaches maximum signal at a lower concentration than 3F11. An ELISA measures endpoint binding while SPR is in real time. The differences in *k*_a_ (on rate) and *k*_d_ (off rate) of the Fab may explain this discrepancy.

**Table 1:** Kinetic constants and binding affinities of the Fab for DENV4 NS2B_18_NS3 full-length.

Fab	*k*_a_ * (M^−1^s^−1^)	*k*_d_ (s^−1^)	*K*_D_ (nM)
3F4	4.03×10^4^	3.95×10^−3^	98.0
3F7	1.75×10^5^	1.65×10^−2^	94.9
3F8	3.18×10^5^	3.34×10^−3^	10.5
3F10	7.21×10^4^	4.83×10^−2^	670.0
3F11	8.48×10^5^	8.02×10^−2^	95.6

*The constants were derived from the sensorgrams in Figure 3B using a simple 1∶1 binding model.

### Effect of the Fab on NS3 enzyme activity

**Figure 4:**
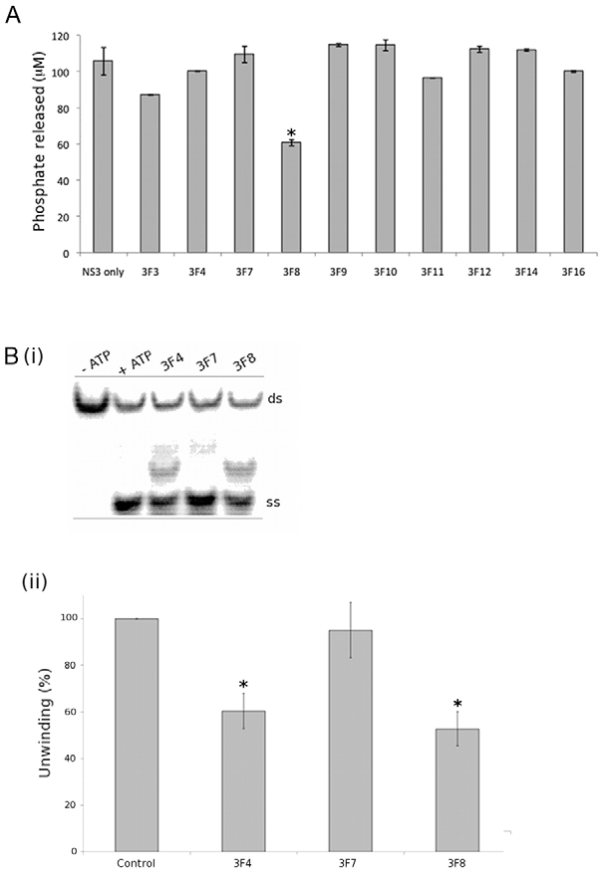
Effect of the Fab on NS3 enzyme activity. Statistical significance (p<0.05) is denoted with an asterix. (A) Comparison of inhibition of DENV4 NS2B_18_NS3 full-length ATPase activity by the Fab. The amount of inorganic phosphate released was measured with the malachite green reagent. (B) Unwinding activity of DENV4 NS2B_18_NS3 full-length using a radio-labelled substrate. The first lane is the negative control in the absence of ATP. Results are expressed as a percentage of the positive control and represent an average of three experiments.

DENV NS3 utilizes ATP to drive the unwinding of the RNA duplex during replication. To determine the inhibitory characteristics of the Fab, DENV4 NS2B_18_NS3 full-length was pre-incubated with 1 µM of each Fab for 30 minutes at room temperature and ATP hydrolysis was monitored in a colorimetric assay [7]. Of the 10 Fab tested, 3F8 was the only antibody that significantly (p<0.05) reduced the amount of phosphate released (60.6±1.7 µM) compared with NS3 alone (105.6±7.6 µM) as seen in Figure 4A.

Protease activity was examined with a DENV2 NS2B_47_NS3pro_185_ construct which contains the 47 amino acids from NS2B sufficient for activating the protease domain. 3F10 inhibits DENV2 protease activity in a dose-dependent manner with activity significantly reduced to 49.5% of control at 1.2 µM (Figure S1 in Supporting Information S1). The epitope for 3F10 is contained within the 18 residues of NS2B that form an N-terminal β-strand distal from the NS3 protease active site (Figure 2A) suggesting allosteric inhibition of the NS2B-NS3 protease.

Helicase assays were performed with a ^32^P-labelled DNA:RNA duplex prepared by annealing an 18-mer DNA oligonucleotide with a 32-mer RNA oligonucleotide. As seen in Figure 4B, with an ATP concentration of 5 mM and a 100-fold molar excess of NS3 relative to nucleotide duplex a significant level of unwinding was observed, in keeping with results published previously [7], [22]. The effect of 3F4, 7 and 8 (the three cross-reactive, helicase specific Fab) on unwinding was assessed at a NS3:Fab molar ratio of 1∶3.5. Both 3F4 and 3F8 significantly (p<0.05) reduced NS3 helicase activity (60 and 53% of control, respectively), while 3F7 had no effect on unwinding.

### 3F8 and DENV infected cells

The helicase specific Fab 3F8 was chosen for further characterisation. This Fab has superior affinity to the other helicase specific Fab, and inhibits both the ATPase and unwinding activities of NS3. The cross-reactive ELISA in Figure 2b demonstrates 3F8 reacts with NS3 from DENV1–4. To determine whether the affinity of the cross-reactions is comparable between serotypes an ELISA using serial dilutions of 3F8 was performed (Figure S2 in Supporting Information S1). The binding curves for DENV1, 2 and 4 overlay while the curve for DENV3 is shifted to the right suggesting lower reactivity with this serotype. A similar trend was seen by SPR where 3F8 was immobilised using amine coupling and kinetic rates and affinity constants were measured for NS3 from the four serotypes (Table S2 in Supporting Information S1). The affinity of DENV3 NS3 was lower (K*_D_* 38.0 nM) than for NS3 from the other serotypes (DENV1 K*_D_* 6.6 nM, DENV2 11.0 nM, DENV4 16.7 nM). Nevertheless, 3F8 binding is in the nanamolar range across serotypes. Western blots were performed to demonstrate 3F8 specifity. The Fab recognises a band of the expected molecular weight of NS3 (70 kDa) in DENV2-infected C6/36 cells but not in uninfected cells demonstrating the detection is robust and specific (Figure S2 in Supporting Information S1).

**Figure 5:**
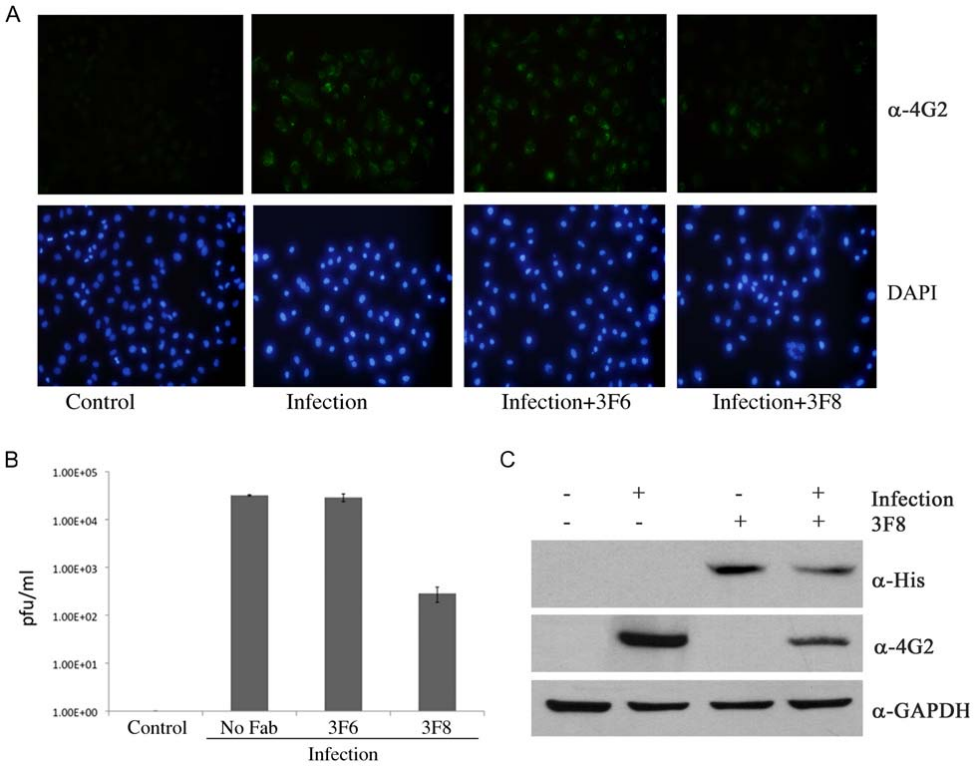
Effect of 3F8 on DENV replication in HEK293 cells. (A) Immunofluorescence using 4G2 mouse monoclonal antibody which stains the DENV envelope protein. The upper panel shows 4G2 images, while lower panel shows DAPI images. (B) Plaque assay performed on culture supernatants 48 hours post infection. (C) Western blot of cells infected with DENV2 probed with anti-His, anti-DENV (4G2) or anti-GAPDH antibody. The figure shows representative blots from two separate experiments.

As NS3 is an intracellular viral enzyme, to observe the effects 3F8 has on DENV replication it must first be delivered across the cell membrane. A protein transfection reagent was used to ensure sufficient 3F8 cell penetration is achieved in HEK293 cells. As shown in Figure 5A, immunofluorescence with the dengue antibody 4G2 shows punctate staining in HEK293 cells infected with DENV2, indicating that 48 hour post-infection most cells were infected with dengue virus where as control cells show no staining. Cells that were transfected with 3F8 prior to infection with DENV2 (Infection +3F8) show less staining for dengue suggesting reduced viral replication in these cells compared with mock transfected cells, and cells transfected with a non-binding Fab control (Infection +3F6). To confirm this plaque assays were performed with supernatants from infected cultures. Cells transfected with 3F8 prior to DENV2 infection showed a two-log decrease in released virus compared with DENV2 infected cells and cells infected with control Fab. Uninfected control cells show no viral replication (Figure 5B). Western blot shows bands in cells transfected with 3F8 using an anti-His antibody indicating successful transfection of His-tagged Fab. A western blot with the anti-dengue 4G2 antibody shows cells transfected with 3F8 have decreased band density verifying the immunofluorescence and plaque assay results (Figure 5C).

### Epitope mapping of 3F8

Peptide inserts obtained after three rounds of panning with the Ph.D-12 random dodecapeptide library and 3F8 were sequenced. Sequences were obtained for twenty phage clones and compared to the sequence for DENV2 NS3 helicase. The DENV2 serotype sequence was chosen for comparison as the peptide array used in the competetion ELISA (described below) was DENV2. Despite using solution panning methods to minimise plastic binding phage, and alternating the affinity resin (between anti-*c-myc* and Ni-NTA resin) to minimise resin binding phage, a large proportion of target-unrelated peptides were obtained [25]. Eighteen of the twenty sequences were highly hydrophobic and the phage clones had no affinity for 3F8 in an ELISA. However, one clone showed sequence identity to DENV2 NS3 helicase. The 12-mer peptide sequence was DETPMRGETRKV, the residues with identity to NS3 helicase are underlined. A second clone also displayed sequence identity with DENV2 NS3 helicase although there were fewer matching residues coompared with the first clone (LSPVQRNNVAII). Both clones had affinity for 3F8 in a phage ELISA and represent possible 3F8 epitopes (data not shown).

**Figure 6:**
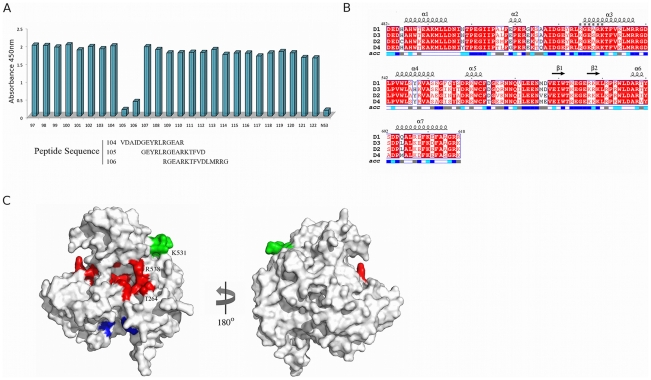
Epitope mapping of 3F8. (A) Competition ELISA results with 3F8 and an array of overlapping 15-mer peptides from subdomain III of DENV2 NS3 helicase. (B) An alignment of NS3 helicase domain III for DENV 1–4. Residues comprising the 3F8 phagotope are highlighted (*) and the accessibility of each residue, derived from the DENV2 NS3 helicase structure (PDB accession code 2BMF), is indicated; blue is highly accessible, cyan is intermediate, white is buried and grey is not predicted. The aligment was performed using ClustalW [32]. (C) Structure of DENV2 NS3 helicase (2BMF) rendered in surface representation. Residues involved in RNA binding are shown in red and the ATP binding site is in blue. Residues corresponding to the 3F8 epitope are shown in green.

To determine which peptide-phage sequence (phagotype) contained the true epitope a competition ELISA was perfomed using an array of overlapping 15-mer peptides from DENV2 NS3 helicase subdomain III. The first phagotype was contained within peptide 105 and the second phagotype within peptide 113. As shown in Figure 6A, peptides 105 and 106 strongly compete with 3F8 for binding to DENV2 NS2B_18_NS3, whereas peptide 113, nor any other peptide from subdomain III, do not compete. The sequences for peptides 104, 105 and 106 suggest the lysine (K_531_) in the RGExRK motif identified by peptide phage display is essential for 3F8 binding as peptide 104 terminates at R_530_ and does not compete in the ELISA.

The 3F8 epitope identified maps to residues 526–531 in the third α-helix (α3′′) in subdomain III of NS3 helicase. An alignment of subdomain III from the four Dengue serotypes (Figure 6B) shows the epitope residues are strictly conserved with the exception of R_526_ which is replaced with a similar, positively charged residue (K_526_) in DENV3. Interestingly, this conservative substitution appears to have some effect on 3F8 reactivity. Signals in western blot and ELISA are lower for NS3 from DENV3 (Figure S3 in Supporting Information S1), and the affinity of 3F8 for DENV3 NS3 was reduced compared with the other serotypes (Table S2 in Supporting Information S1).

Surface accessibity derived from an apo DENV2 NS3 helicase crystal structure [7] is shown underneath the alignment in Figure 6B. Of the residues in the RGExRK motif R_526_, G_527_, E_528_ and K_531_ are highly accessible (blue) while R_530_ is buried (white) suggesting it may not interact with 3F8 in the structural epitope. The position of the epitope (green) on the surface of DENV2 NS3 helicase is shown in Figure 6C. It is distal from the ATP binding site (blue) but is in close proximity to residues involved in RNA binding (red). The ATP and RNA binding sites were previously proposed and/or observed in the DENV2 and DENV4 crystal structures [7]. The distance between the α-carbons of K_531_ in the 3F8 epitope and R_538_ and T_264_ in the RNA binding tunnel is 10.5 and 14.9 Å, respectively.

## Discussion

A naïve human Fab-phage library has been successfully employed to isolate antibody fragments with specificity for DENV4 NS2B-NS3. They can be broadly grouped based on their domain specifity; those that bind the NS3 helicase domain (3F3, 4, 7, 8 and 16), those that bind the 18 residues of NS2B required for correct folding and stability of the protease domain (3F10 and 11), and a final Fab that binds the 10 residue linker between the protease and helicase domains of NS3 (3F9). The helicase domain appears to have dominated the selection process with the majority of Fab binding this domain. This is most likely due to the size of the helicase domain (50 kDa) relative to that of the protease domain (20 kDa), rather than a lack of epitopes on the protease domain since a recent study in which the NS3 protease domain from West Nile Virus was subject to phage display identified several protease specific Fab [26], albiet with a synthetically expanded library.

From the 10 antibodies originally isolated, three of the helicase specific Fab (3F4, 7 and 8) cross-react with NS3 from the four DENV serotypes (Figure 2B). Of these, 3F8 was the most promising for detailed characterisation as it binds NS3 with 10-fold higher affinity (K*_D_* 10.5 nM) compared with 3F4 (K*_D_* 98.0 nM) and 3F7 (K*_D_* 94.9 nM). The epitope of 3F8 has been mapped to residues 526–531 in subdomain III of the helicase domain (Figure 6C). Subdomains I and II are observed across the SF2 superfamily of helicases. However subdomain III is unique to the flaviviruses [7] and it has been shown to influence DENV NS3 activity, with mutation of a single arginine (Arg_513_) to alanine in α2″ producing a defective helicase [27]. An alignment of flavivirus NS3 sequences shows the 3F8 epitope (RGExRK) is essentially conserved across several members of the flaviviridae including Japanese encephalitis virus, West Nile virus, Tick Borne encephalitis virus and Yellow fever virus suggesting the antibody will cross react with NS3 from these species (Figure S3 in Supporting Information S1). However, the epitope is not observed in hepatitis C virus, and the largest differences in both sequence and structure between DENV and hepatitis C virus NS3 have been observed in subdomain III [7].

The 3F8 epitope is distal from the ATP binding pocket, and 10–15 Å from the subdomain I end of the RNA tunnel. The impact 3F8 has on NS3 enzymatic activities was assessed using biochemical assays. 3F8 reduces NS3 catalysed unwinding of a double stranded DNA:RNA substrate (Figure 4C). When 3F8 is bound, accessibility to the RNA binding site may be reduced, especially when the size of a Fab molecule (50 kDa) is considered. Allosteric effects induced by 3F8 binding may also contribute to the reduced activity observed. Large quaternary changes have been observed in NS3 upon RNA binding [18] and these may be hindered in the presence of a strongly binding subdomain III antibodies such as 3F8. The helicase and ATPase activities of NS3 are linked with ATP hydrolysis being induced by RNA binding. This drives translocation of NS3 in a 3′ to 5′ direction along an RNA substrate. Interestingly, 3F8 was the only antibody to reduce ATP turnover by NS3 (Figure 4A). It is possible 3F8 binding induces a conformational change in the distal ATP binding pocket, or alternatively it may restrict RNA binding and, in turn, reduce ATP turnover. The ATPase assay includes poly(U) to stimulate NS3 ATPase activity [28].

The reduced enzyme activity observed in biochemical assays translated to a cell-based system with 3F8 reducing DENV replication in HEK293 cells (Figure 5B). While the direct effect 3F8 has on NS3 activity will contribute to this reduced replication, protein-protein interactions must also be considered. As part of the viral replicative complex, NS3 interacts with the RNA-dependent RNA polymerase NS5. The region for the NS5 interaction has been mapped to subdomains II and III of NS3 [29], and 3F8, by binding subdomain III may interfere with this interaction. NS3 co-localises with the integral membrane protein NS2B in infected cells [30] and Fab binding may also have steric effects in this context, inhibiting protein rearrangments that occur in close proximity with the endoplasmic reticulum membrane during the replication cycle. More studies are required to understand how the various DENV proteins assemble to form the molecular machinery necessary for RNA replication, and a panel of epitope mapped, NS3 and NS5 specific antibodies such as 3F8 may prove powerful tools in such studies.

The high specificity and affinity of 3F8, together with its ability to inhibit DENV replication may be further exploited in therapeutics. However as NS3 activity is intracellular, transport through the cell membrane is a major hurdle in developing anti-NS3 antibodies for clinical use. Experimental approaches such as coupling the antibody with a transport protein [31], or expressing the antibody fragment as an intracellular protein (intrabody) using gene therapy vectors maybe considered to overcome the challenges associated with delivery of biomolecules into cells.

## Supporting Information

Supporting Information S1 Figure S1. Inhibition of DENV2 NS2B_47_NS3pro_185_ protease activity by 3F10. Figure S2. Cross-reactivity of 3F8 with NS3 from DENV1-4 as measured by ELISA and western blot. Figure S3. Sequence alignment of amino acids spanning the 3F8 epitope in flavivirus NS3 proteins. Table S1. Sequence analysis and variable gene usage of anti-NS3 Fab. Table S2. Kinetic constants and binding affinities of 3F8 with NS3 from DENV1-4 determined by surface plasmon resonance.
(0.17 MB DOC)
