# Supplementary material for: High Affinity Human Antibody Fragments to Dengue Virus Non-Structural Protein 3
Source: PLoS Negl Trop Dis. 2010 Nov 9;4(11):e881. doi: 10.1371/journal.pntd.0000881 (PMC2976680; doi:10.1371/journal.pntd.0000881)
Supplement: Supporting Information S1 — Figure S1. Inhibition of DENV2 NS2B47NS3pro185 protease activity by 3F10. Figure S2. Cross-reactivity of 3F8 with NS3 from DENV1-4 as measured by ELISA and western blot. Figure S3. Sequence alignment of amino acids spanning the 3F8 epitope in flavivirus NS3 proteins. Table S1. Sequence analysis and variable gene usage of anti-NS3 Fab. Table S2. Kinetic constants and binding affinities of 3F8 with NS3 from DENV1-4 determined by surface plasmon resonance. (0.17 MB DOC) [file pntd.0000881.s001.doc]

**Supporting Information S1**

**Figure S1.** Inhibition of DENV2 NS2B_47_NS3pro_185_ protease activity by 3F10. While both 3F10 and 3F11 bind NS2B, only 3F10 cross-reacts with DENV2 (Figure 2B) so protease inhibition was assessed for this Fab. Fab were preincubated with 10nM DENV2 NS2B_47_NS3pro_185_ for 30 minutes at room temperature. The 30 μl reaction contained 50mM Tris pH 8.5, 20% glycerol, 1mM CHAPS and 20 μM of substrate (Bz-Nle-Lys-Arg-Arg-AMC). Following an incubation of 30 minutes at 37°C the released AMC fluorophore was monitored at a λ_ex_ of 380nm and an λ_em_ of 450 nm. Data were normalised against a no Fab control and data points significantly reduced compared with control (p < 0.05) are denoted with an asterix.

**
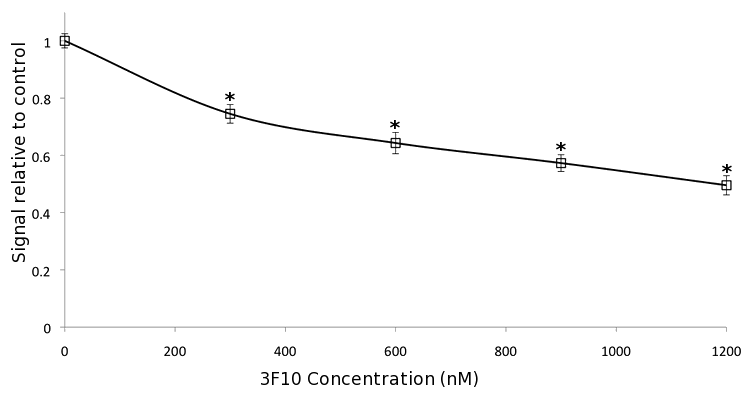
**

**Figure S2.** Cross-reactivity of 3F8 with NS3 from DENV1-4. (A) Comparison of binding to NS3 full-length proteins from all four dengue serotypes (DENV-1 Hawaii, DENV-2 TSV01, DENV-3 S22103 and DENV-4 MY22713) as measured by ELISA. (B) Western blot recognition of DENV NS3 by 3F8. For bacterial cultures 200 ng of purified NS2B_18_NS3 full-length was added to *E. coli* soluble protein fraction (15 μg of total protein) for analysis. C6/36 cells were infected at a MOI of 1.0 with DENV2 (Eden 3295) and cultured for 3 days. Equal amounts of C6/36 lysate were loaded for SDS‑PAGE. Blots were probed with 3F8 (1nM) followed by detection with an HRP conjugated goat anti‑human IgG heavy and light chain (MyBioSource).

**
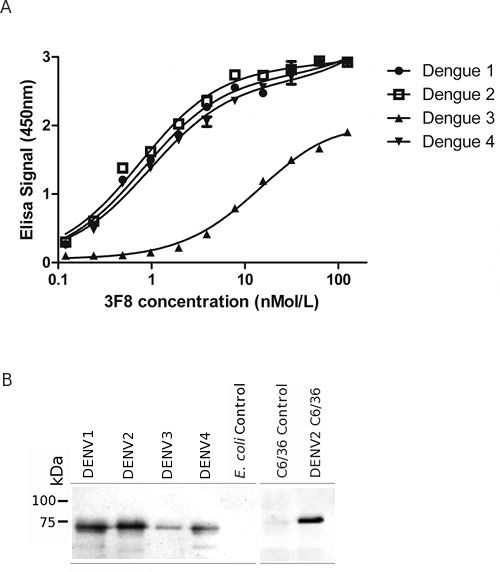
**

**Figure S3.** Sequence alignment of amino acids spanning the 3F8 epitope in flavivirus NS3 proteins. The DENV sequence is derived from the DENV-2 NS2B_18_NS3 construct used in this study (strain TSV01). The sequences of Japanese encephalitis (JEV, NP_059434), West Nile Virus (WNV, AAT02759), tick-borne encephalitis (TBV, NP_043135), yellow fever virus (YFV, NP_041726) and hepatitis C virus (HCV, NP_671491) were obtained from GenBank. The epitope motif (RGExRK) for 3F8 is highlighted in yellow. The alignment was performed with T-Coffee (www.tcoffee.org).

DENV EKVDAIDGEYRLRGEARKTFVDLMRR--GDLPVWLAYR---V

JEV EKAFTMDGEYRLRGEEKKNFLELLRT--ADLPVWLAYK---V

WNV EKVYTMDGEYRLRGEERKNFLEFLRT--ADLPVWLAYK---V

TBV DKMPEVAGHFRLTEEKRKHFRHLLTH--CDFTPWLAWH---V

YFV TKTPVSPGEMRLRDDQRKVFRELVRN--CDLPVWLSWQ---V

HCV DHLEFWEGVFTGLTHIDAHFLSQTKQSGENFPYLVAYQATVC

: * . * ::. ::::

**Table S1.** Sequence analysis and variable gene usage of anti-NS3 Fab. Shown are the complementary determining region 3 (CDR3) and variable gene family designations, assigned using IMGT/V-Quest (http://imgt.cines.fr/).

| Fab Clone | CDR3 V_L_ | V_L_ family | CDR3 V_H_ | V_H_ family |
| --- | --- | --- | --- | --- |
| NS2B_18_ |  |  |  |  |
| 3F10 | QQRYNWPPIT | Vκ3 | ARVPILLSGDAFDI | VH3 |
| 3F11 | MQGTHWPLLT | Vκ2 | ARIPKASIVWG | VH3 |
|  |  |  |  |  |
| NS3 helicase |  |  |  |  |
| 3F3 | MQGLQTPRT | Vκ2 | ATLRGYYYMDV | VH3 |
| 3F4 | QSYDSSSVV | Vλ6 | AREYSSSWHYGMDV | VH3 |
| 3F7 | QSYDSSSVV | Vλ6 | ARDHGYSYGYFDY | VH3 |
| 3F8 | QSYGSTSYV | Vλ6 | ARDDGGDAFDI | VH4 |
| 3F16 | QSYDSRTQKWV | Vλ6 | ASLGYYDSSGYYFDY | VH3 |
|  |  |  |  |  |
| Inter-domain linker |  |  |  |  |
| 3F9 | ASWDDSLNAWV | Vλ1 | ARGWELLHY | VH3 |
|  |  |  |  |  |
| Full-length NS3 only |  |  |  |  |
| 3F12 | HSFDIRLSADI | Vλ1 | ARVGPGDLWFGDPLDY | VH4 |
| 3F14 | SSYAANNKVL | Vλ2 | ARLLPFWWSPAFDI | VH4 |
|  |  |  |  |  |

**Table S2.** Kinetic constants and binding affinities of 3F8 for NS2B_18_NS3 full‑length proteins from DENV1-4 determined by surface plasmon resonance. 3F8 was covalently immobilized on a CM5 chip using amine coupling chemistry. Kinetic paramters were measured by varying the molar concentration of each NS3 protein (18.75–300nM).

| **Fab** | ***k*_a_*(M^-1^s^-1^)** | ***k*_d_ (s^-1^)** | ***K*_D_ (nM)** |
| --- | --- | --- | --- |
| D1 (Hawaii) | 6.5 x 10^4^ | 2.3 x 10^-4^ | 6.6 |
| D2 (TSV01) | 6.8 x 10^4^ | 7.4 x 10^-4^ | 11.0 |
| D3 (S22103) | 2.7 x 10^4^ | 2.2 x 10^-3^ | 38.0 |
| D4 (MY22713) | 7.3 x 10^4^ | 1.2 x 10^-3^ | 16.7 |

*The constants were derived using a simple 1:1 binding model
